# Supplementary material for: Production of Phytotoxic Metabolites by Botryosphaeriaceae in Naturally Infected and Artificially Inoculated Grapevines
Source: Plants (Basel). 2021 Apr 19;10(4):802. doi: 10.3390/plants10040802 (PMC8073839; doi:10.3390/plants10040802)
Supplement: Supplementary file 1 [file plants-10-00802-s001.zip › plants-1171663-supplementary.pdf]

Production of phytotoxic metabolites by Botryosphaeriaceae in naturally-infected and artificially-inoculated grapevines

Pierluigi Reveglia[1,2,3*], Regina Billones-Baaijens[1], Jennifer Millera Niem[1,4], Marco Masi[2], Alessio Cimmino[2], Antonio Evidente[2], Sandra Savocchia[1]

[1]*National Wine and Grape Industry Centre, School of Agricultural and Wine Sciences, Charles Sturt University, Locked Bag 588, Wagga Wagga, NSW 2678, Australia.*
[2]*Dipartimento di Scienze Chimiche, Universita' di Napoli Federico II, Complesso Universitario Monte Sant'Angelo, Via Cintia 4, 80126 Napoli, Italy.*
[3]*Current Address: Department of Clinical and Experimental Medicine, University of Foggia, Viale Pinto 1, 71121, Foggia, Italy.*
[4]*Current Address: UPLB Museum of Natural History, University of the Philippines - Los Baños, College, Laguna, 4031 Philippines.*
* Corresponding author: pierluigi.reveglia@unifg.it

Supporting information list

Figure S1. Representative photos of the inoculated vines showing internal staining of the wood at (a) 6 months; b) 12 months post-inoculation with *Diplodia seriata* H141a; *Spencermartinsia viticola* DAR78870; *Dothiorella vidmadera* DAR78993; and non-inoculated control.

Figure S2. Representative LC-MS/MS chromatograms of naturally-infected wood extracted using two extraction protocols: a) MRM chromatogram of Riverina vine 3 extracted with protocol B (red) and protocol A (green); b) MRM transitions at retention time 27.44 correspond to $[M + H]^+$ 179.1 m/z precursor ion of (*R*)-mellein.

Figure S3. LC-MS/MS analysis of Riverina vines: a) MRM chromatograms of Riverina's symptomatic wood sample from vine 1 (green), vine 2 (purple), vine 3 (red) and Riverina asymptomatic wood sample (black); b) MRM transitions at retention time 27.44 corresponding to $[M + H]^+$ 179.1 m/z precursor ion of (*R*)-mellein; c) Number of copies of pathogen DNA quantified by qPCR in Riverina vines.

Figure S4. MRM Chromatograms of Chardonnay vine inoculated with *D. seriata* H141a 12 months post inoculation (red) and Chardonnay vine inoculated with *N. parvum* DAR78998 6 months post inoculation (green).

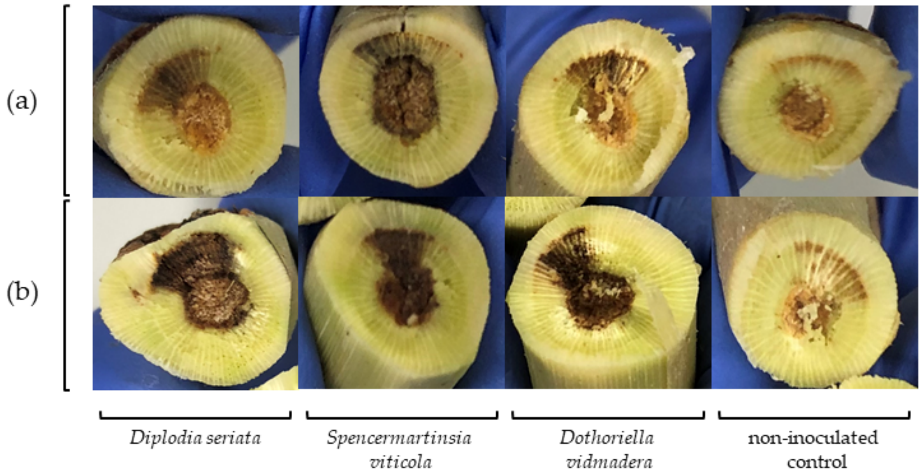

Figure S1. Representative photos of the inoculated vines showing internal staining of the wood at (a) 6 months; b) 12 months post-inoculation with *Diplodia seriata* H141a; *Spencermartinsia viticola* DAR78870; *Dothiorella vidmadera* DAR78993; and non-inoculated control.

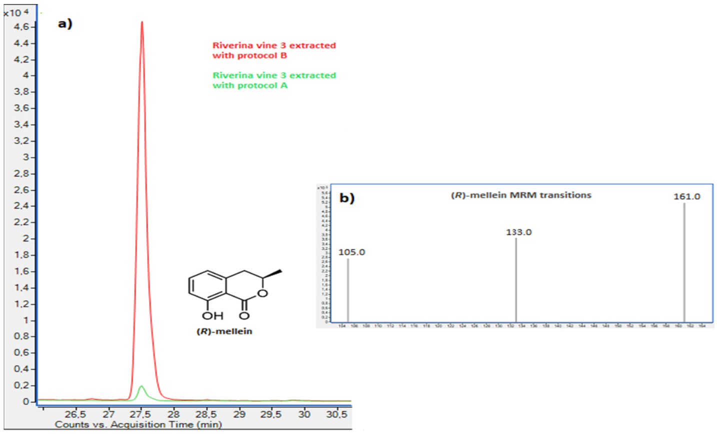

Figure S2. Representative LC-MS/MS chromatograms of naturally-infected wood extracted using two extraction protocols: a) MRM chromatogram of Riverina vine 3 extracted with protocol B (red) and protocol A (green); b) MRM transitions at retention time 27.44 correspond to $[M + H]^+$ 179.1 m/z precursor ion of (*R*)-mellein.

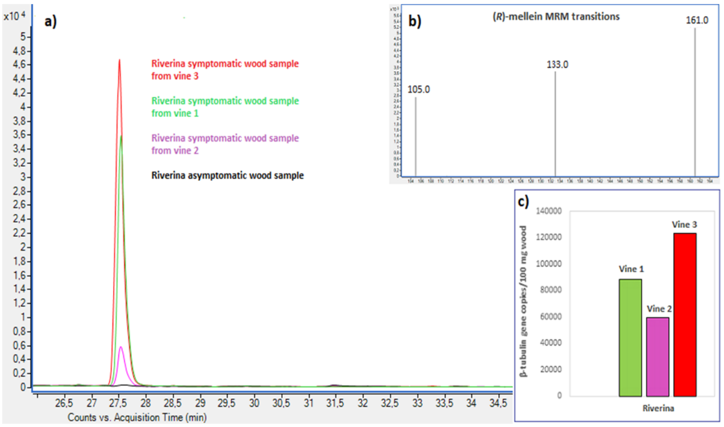

Figure S3. LC-MS/MS analysis of Riverina vines: a) MRM chromatograms of Riverina's symptomatic wood sample from vine 1 (green), vine 2 (purple), vine 3 (red) and Riverina asymptomatic wood sample (black); b) MRM transitions at retention time 27.44 corresponding to $[M + H]^+$ 179.1 m/z precursor ion of (*R*)-mellein; c) Number of copies of pathogen DNA quantified by qPCR in Riverina vines.

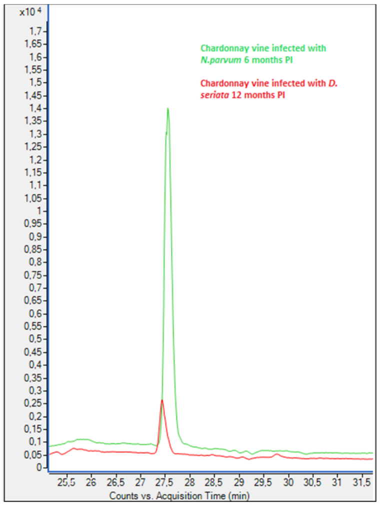

Figure S4. MRM Chromatograms of Chardonnay vine inoculated with *D. seriata* H141a 12 months post inoculation (red) and Chardonnay vine inoculated with *N. parvum* DAR78998 6 months post inoculation (green).
